# Editorial: Cardiovascular physiology and pathology of cardio-pulmonary and peripheral sensory nerves

**DOI:** 10.3389/fphys.2023.1188800

**Published:** 2023-03-31

**Authors:** Irving H. Zucker, Neil Herring, Han-Jun Wang

**Affiliations:** ^1^ Department of Cellular and Integrative Physiology, University of Nebraska Medical Center, Omaha, NE, United States; ^2^ Burdon Sanderson Cardiac Science Centre, Department of Physiology, Anatomy and Genetics, University of Oxford, Oxford, United Kingdom; ^3^ Department of Anesthesiology, University of Nebraska Medical Center, Omaha, NE, United States

**Keywords:** sympathetic, autonomic, vagus, sensory, cardiovascular reflex, chemoreflex

This editorial summarizes a Research Topic that is, critical to our understanding of the neural control of cardiopulmonary function. It is well known that the cardiopulmonary system is endowed with a variety of sensory receptors that are necessary for cardiovascular homeostasis. For the most part, stretch receptors in the heart, lungs and great vessels participate in classical negative feedback reflexes to regulate blood pressure, blood volume and in the case of chemoreceptors oxygen and carbon dioxide tension. The peripheral and central pathways of many of these receptors are vagal or glossopharyngeal. On the other hand, there are a separate group of receptors whose axons enter the central nervous system through the dorsal root ganglia accessing the spinal cord and ascending through the dorsal columns to evoke excitatory responses, especially *via* the sympathetic nervous system. The latter input often activates a positive feedback state and is considered to be enhanced by noxious stimuli in pathological states (e.g., following ischemia). Many of these spinal afferents transmit sensations of pain emanating from the heart and referred areas during coronary ischemia. In this regard the review article by Jiang et al. is highly relevant. These authors point out the importance of TRPV1 and TRPA1 signaling in the genesis of cardiac pain in sympatho-excitation following myocardial ischemia (see their figure 3).

Post-ganglionic sympatho-excitation (stellate ganglia) that would occur in hypertension and heart failure is mediated by major alterations in ion channel activity. In this regard the study by Davis et al. is an important contribution. These authors used whole cell patch clamp recordings to show a mechanism by which these neurons can sense extracellular Na^+^ through the Na_x_ channel. This mechanism may play a role in coupling plasma osmolality to changes in sympathetic outflow, a novel idea that requires further study.

The discharge sensitivity from sensory endings from the thoracic vagi is regulated by many factors that ultimately modulate ion channel function and membrane potential. Of course, vagal reflexes can be impacted in a major way by external substances. In a novel study by Fioretti et al. they investigated the effects of Vitamin D on cardiopulmonary reflexes. This Research Topic is relevant to both environmental exposure to ultraviolet radiation and to excessive dietary intake of Vitamin D. Rats supplemented with Vitamin D showed exaggerated vagal reflexes activated by phenybiguanide and showed enhanced bradycardic responses to activation of the arterial baroreflex. This study points to a potential hazardous effect of Vitamin D supplementation, currently widely used in the human population.

Chemoreflexes mediated by receptors in the carotid body and aortic arch are classically activated by hypoxia and/or by stagnant anoxia if carotid body blood flow is reduced. An increased sensitivity of the peripheral chemoreflexes has been demonstrated in the heart failure state (Andrade et al., 2018) however, it is not clear if a similar change takes place after acute lung injury. In a study by Kamra et al. acute lung injury was induced by intra-tracheal administration of bleomycin in rats and chemoreflex sensitivity examined over time following lung injury. Interestingly, the response to hypoxia was slightly exaggerated in the early stage following lung injury but was significantly sensitized during the recovery stage at approximately 4 weeks post injury. The implication of this study relates to a potential for enhancement of sympathetic outflow during late-stage recovery from lung injury and the potential initiation of cardiac arrythmias. The molecular mechanisms responsible this phenomenon remains to be elucidated. A follow-up study by Kamra et al. further explored the potential neural mechanisms underlying chronic chemoreflex sensitization during the recovery of lung injury. They reported that the superior cervical ganglia (SCG) are involved in the chronic chemoreflex sensitization process since surgical removal of SCG (SCGx) prevented either hypoxia- or hypercapnia-evoked increase in respiratory rate in rats during the recovery of lung injury.

Under this Research Topic an interesting and potentially important study was carried out by Huo et al. The exercise pressor reflex (EPR) originates from mechano and metabo-receptors in skeletal muscle usually when blood flow is compromised during exercise (review). This results in an exaggerated sympathetic response and an increase in arterial pressure. Several pathological states cause an increase in the sensitivity of this reflex. This includes chronic heart failure (Wang et al., 2015), and type 2 diabetes mellitus (T2DM) Huo et al. In this study the enhancement in the EPR in T2DM was separated from aging *per se* since T2DM and aging are often correlated. These investigators showed a clear separation between T2DM and aging in the enhanced metabo- and mechano-responses to hindlimb muscle contraction in rats. While aging is associated with several metabolic abnormalities in skeletal muscle, this study shows that it does not seem to be a factor in the exaggerated EPR in T2DM.

In a final review in this series of papers, Yu promotes the notion of Multiple Sensory Theory (MST). This concept helps to understand a variety of sensory signaling modalities in the cardiovascular system. One axon may give rise to multiple sensory endings located in disparate areas of the circulation. Furthermore, one receptor may code for multiple changes in the substrate that they are embedded in and integrate input from multiple endings. This review not only provides an historical overview of cardiovascular sensory transduction but also promotes the idea that molecular changes at the receptor ending may be responsible for MST.

**FIGURE 1:**
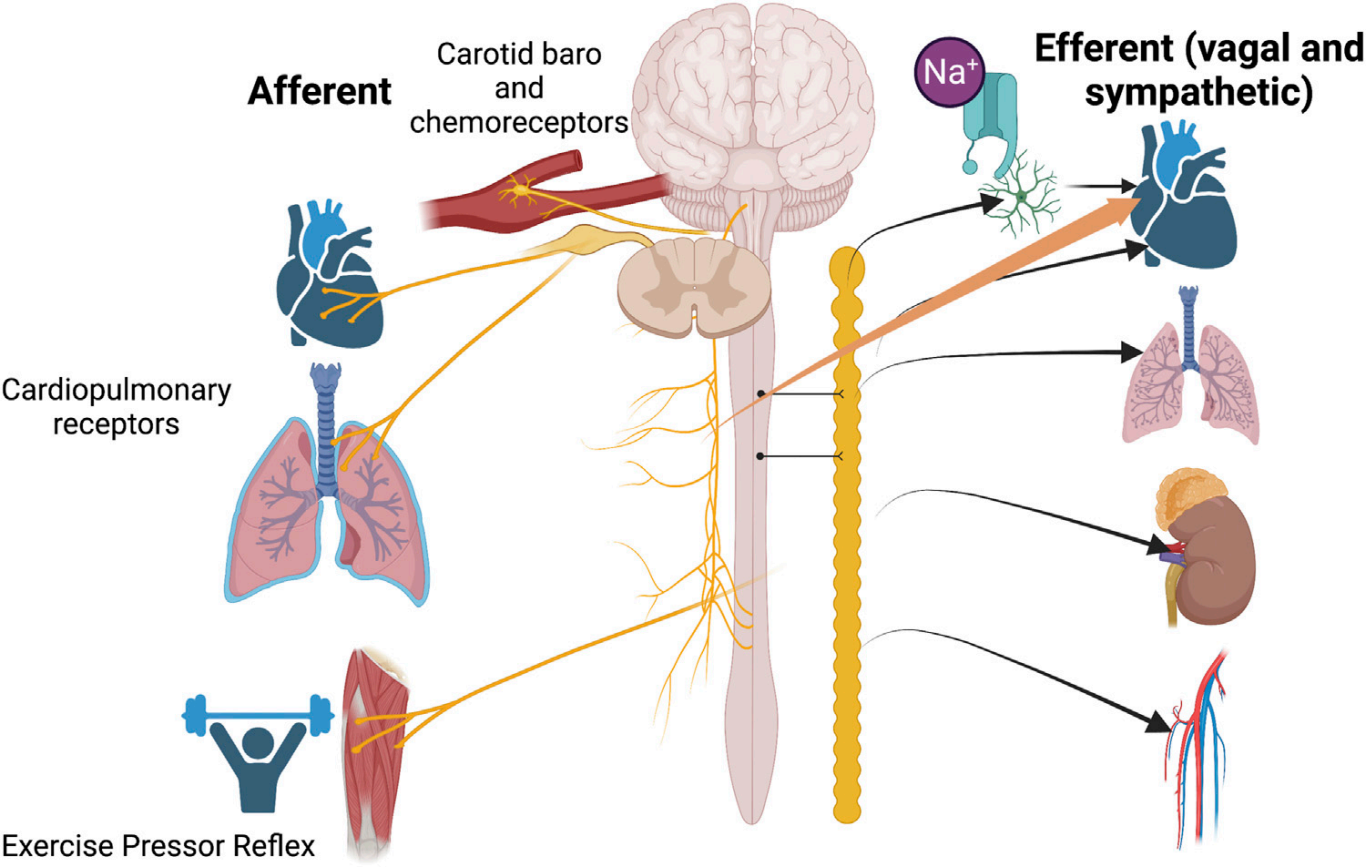
An overview of the contributions of papers in this Research Topic to our understanding of the role played by cardiopulmonary sensory receptors to sympatho-vagal outflow. Each paper in this series focuses on one aspect of afferent input to the central nervous system. Sensory input may be uni or multi-dimensional so that transduction of various modalities including stretch, oxygen tension and nociception is signaled through cardiopulmonary afferents.

Figure 1 incorporates the interplay of sensory transduction in the cardiopulmonary region with concepts proposed in the current Research Topic.
